# Endovascular treatment of primary hepatic tumours

**Published:** 2008-11-15

**Authors:** B Popa, M Popiel, L Gulie, C Turculeţ, M Beuran

**Affiliations:** Clinical Emergency Hospital BucharestRomania

## Abstract

First transcatheter embolization of hepatic artery has been materializing in 1974, in France, for unresectable hepatic tumours. Then, this treatment has become use enough in many countries, especially in Japan, where primary hepatic tumours are very frequent.

In this article, we present procedures of interventional endovascular treatment for primary hepatic tumours: chemoembolization, intra–arterial chemotherapy.

The study comprises patients with primary hepatic tumours investigated by hepatic–ultrasound and contrast–enhanced CT or MRI. DSA–hepatic angiography is very important to verify the accessory hepatic supply. It has been performed selective catheterization of right/left hepatic branches followed by cytostatics injection. Most of the patients have benefit by hepatic chemoembolization (cytostatics, Lipiodol and embolic materials). The selective intra–arterial chemotherapy (cytostatics without Lipiodol) was performing in cases with contraindications for Lipiodol or embolic materials injection (cirrhosis–Child C, thrombosis of portal vein, hepatic insufficiency). For treatment of primary hepatic tumours we use 5–F–Uracil, Farmarubicin and Mytomicin C. Less numbers of the reservoirs were placed because financial causes.

Chemoembolization was better than procedures without Lipiodol or embolic materials. Lipiodol reached in tumoural tissue and the distribution of Lipiodol harmonises with degree of vascularisation. After the chemoembolization procedure, the diameter of tumours decreased gradually depending on the size of tumour.

Effective alternative for unresectable primary hepatic tumours (big size, hepatic dysfunction, and other surgical risk factors) is endovascular interventional treatment.

## Introduction

Hepatocellular carcinoma is the most frequent primary hepatic tumour with an incidence between 80% and 90%. This type of cancer is discovered six to eight times more frequent in men with the greatest risk of appearance from sixth to eighth decade in regions with low incidence but it may appear between thirty to forty years in populations where hepatocellular carcinoma has a higher incidence [1, 14].

A series of factors favour the appearance of hepatocellular carcinoma, hepatic cirrhosis being the most important.

The growing incidence of HBV and HCV infections increased the number of hepatocellular carcinoma associated with viral cirrhosis. Also, alcoholic cirrhosis, more frequent in our country, present a certain malignant risk, based on the carcinogenic effect of alcohol. Another risk 'factors responsible for hepatocellular carcinoma are Aflatoxin B1 (a product of a mould called *Aspergillus flavus*), hemochromatosis, Wilson disease or thyrosinemia [8, 9].

Usually, patients with hepatocellular carcinoma have an atypical simptomathology. Several patients may present diffuse abdominal pain, loss of appetite, bloating, asthenia or cirrhotic symptoms; viral infection is diagnosed in the same time with primitive tumour in most of the cases.

## Screening

**Fig. 1:**
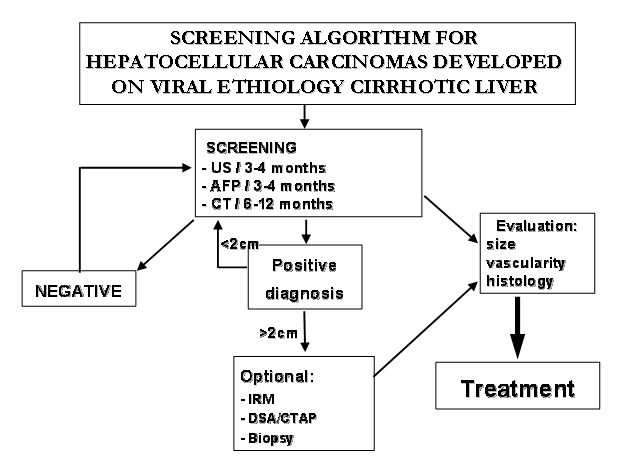
Hepatocellular carcinoma screening algorithm

For the early diagnosis of small (under 2 cm) and asymptomatic hepatocellular carcinoma, screening of patients with chronic hepatic disease has been made with the aid of ultrasonography and the blood values of alfa-fetoprotein, HBV and HCV markers determined on every three months (Fig. 1) [12].

Abdominal ultrasonography is the first intention diagnostic method and it has been used also for monthly evaluation of endovascular treatment. Early hepatocellular carcinoma presents as an unique or multiple nodular tumour with relative well defined borders, parenchyma consistency and hipoechogenic. HCC presents unhomogenous echographic structure and rarely intratumoral calcifications. Ultrasonography may also discover portal trunk or branches thrombosis (often associated with hepatocarcinoma).

Later on, primitive tumours smaller than 2 cm discovered by ultrasonography will be evaluated by abdominal CT. In the areas with high incidence of viral hepatitis, abdominal CT is recommended every 6 to 12 months even if the echography is negative [1, 12]. On native examination, hepatocellular carcinoma presents as a hypodense (in the majority of the cases) or isodense mass with central hypodense (zones or necrosis or fatty liver) or hyperdense areas (recent tumoral haemorrhages). In the arterial phase of the contrast injection CT, HCC is shown as a hiperdense mass with rapid wash-out while in the venous phase in appears hypodense with hiperdense capsule and septa. IRM may be used for the diagnosis of hepatic tumour in patients with cirrhotic liver and regenerative nodules and also in patients whose ultrasonographic exam diagnosed hepatomas larger than 2 cm.

Selective hepatic angiography is used to confirm the diagnosis of hepatic tumour, to better visualize the hepatic arterial vessels, to discover eventual arterial–portal shunts and also to decide which type of endovascular treatment protocol should be followed (chemoembolisation, i.e. chemotherapy or subcutaneous reservoir implantation). Along with a selective hepatic arteriography, an angiography of the celiac trunk is necessary to determine the eventual presence of a tumoral extra–vascularisation (branches from left–gastric, gastro–duodenal or phrenic arteries) or the superior mesenteric arteriography in order to visualize the possible origin of the right hepatic artery from the proximal part of the superior mesenteric artery [2, 3].

## Treatment

**Fig. 2:**
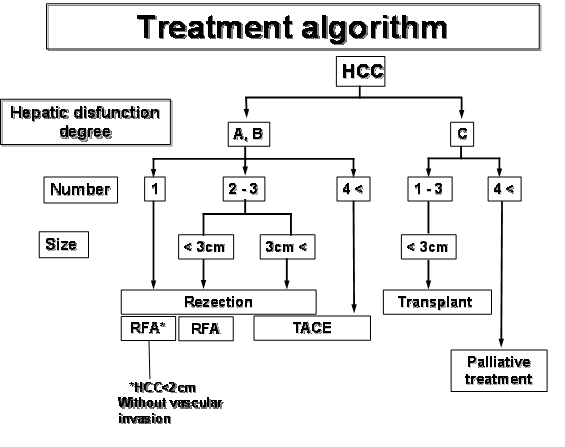
Hepatomas treatment algorithm

The treatment algorithm should always be decided following important data such as the clinical status of the patient, the hepatic dysfunction degree or the tumours' number, size and location (Fig. 2).

Endovascular treatment is mandatory for several situations:

- Unresectable tumours: multiple tumours, tumours greater than 8 cm, vascular invasion or tumours situated too close to major hepatic vessels.
- First two Okuda stage hepatic cirrhosis associated with tumours between 4 cm and 8 cm in diameter [4 , 5].
- Tumours smaller than 4 cm in diameter in which radiofrequency ablation was contraindicated
- Anesthetic or surgical risks.

### Case 1

**Fig. 3:**
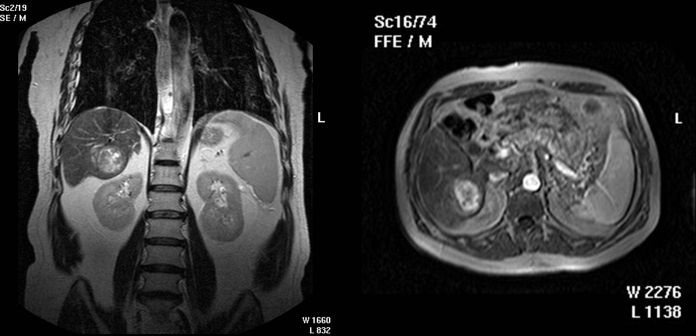
Primitive tumor on cirrhotic liver with hepatitis B

67 years–old patient, without any personal pathology, presents to the hospital having unspecific simptomathology such as fatigue, inapetency or right upper quadrant pain. The ultrasonography describes a cirrhotic liver which presents in the sixth segment a hipoechogenic, unhomogenous tumour of 51mm in diameter. Later on, the diagnosis is sustained by the IRM findings (Fig. 3), the high levels of alfa–fetoprotein and the presence of hepatitis B.

**Fig. 4:**
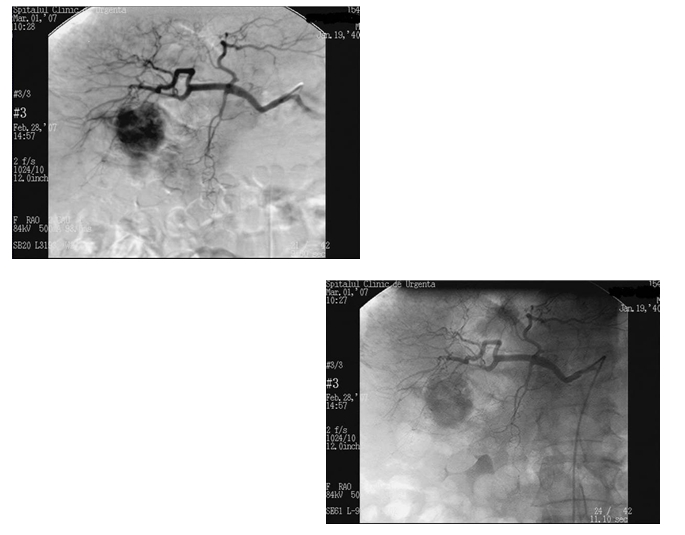
Diagnostic arteriography

The diagnostic arteriography described a hipervascular, unhomogenous, septated, relatively well defined tumour, irrigated from a segmentary branch of the right hepatic artery (Fig. 4). Superior mesenteric arteriography confirms the permeability of the portal vein.

Following the treatment algorithm, is decided that intra–arterial chemo–embolisation is the most suitable treatment method for our case, consisting of monthly sessions of 1 hour perfusion with 5–Fluoro–Uracil 750mg followed by a mixture between Farmarubicin 50mg, Mytomicin C 15mg and Lipiodol. The arterial branch irrigating the tumour was than occluded with TachoComb. After the first chemoembolisation, arteriography showed the absence of contrast inside the tumour while the vascular branch still being permeable.

During intervention, the patient presented mild epigastric pain, remitted after ingestion of analgesics. Other post–chemoembolisation symptoms were nausea and fever.

**Fig. 5:**
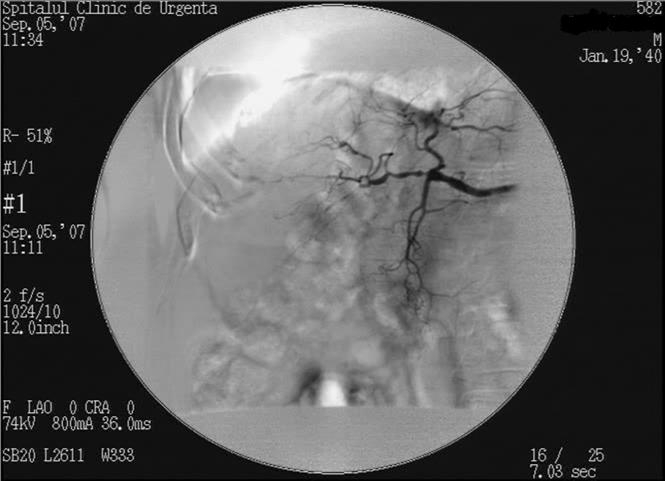
DSA – 9 months post–embolisation

**Fig. 6:**
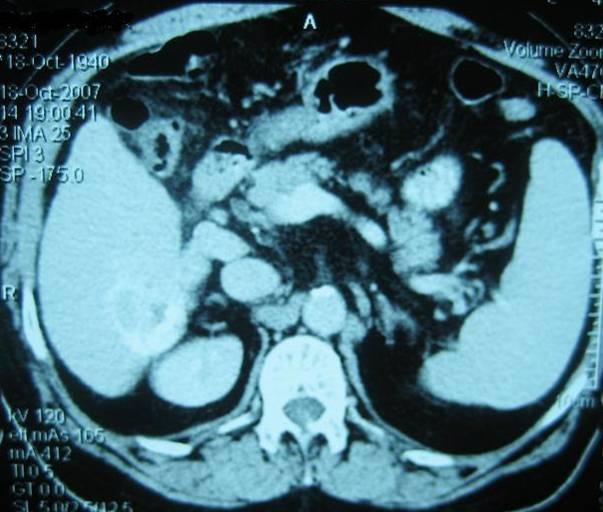
CT – 9 months post–embolisation

After 9 chemoembolisation sessions the AFP was normal and the CT described that the tumour decreased its activity and reduced its dimensions by thirty percent (Fig. 6), situation also confirmed by the arteriography (Fig. 5).

### Case 2

56 years–old patient, diagnosed 3 years ago with hepatitis C, third degree oesophageal varices, following a four months ultrasonographic screening, is diagnosed with hepatoma situated in the fifth hepatic segment.

**Fig. 7:**
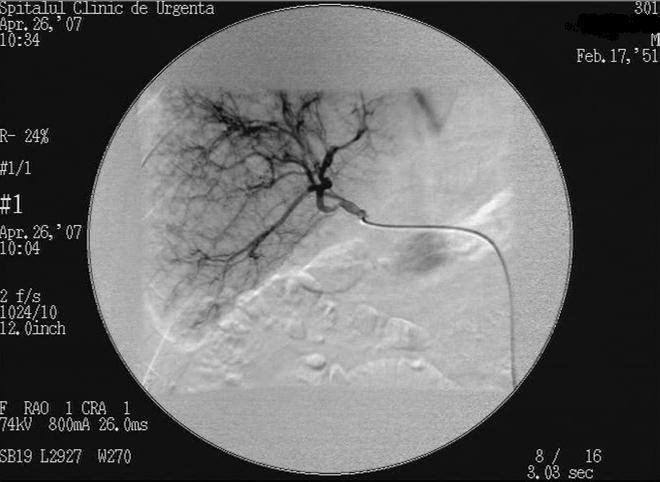
Pre–embolisation DSA

Abdominal CT and angiography confirmed the echographic diagnosis (Fig. 7). Because of the decompensate cirrhosis and the surgical risks, tumour resection is contraindicated, so it's decided that the best treatment solution for our patient is monthly hepatic chemoembolisation sessions with 5%Fluoro%Uracil 750mg, Farmarubicin 50mg, Mytomicin C 15mg, Lipiodol and TachoComb. Fortunately, the patient hasn't developed any post–embolisation syndrome.

**Fig. 8:**
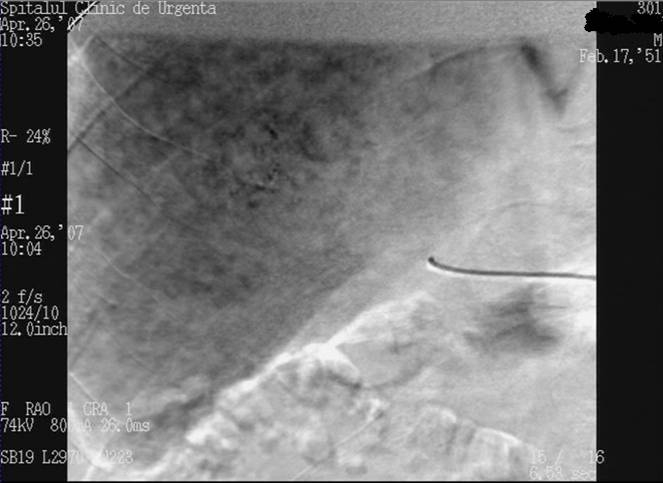
12 months post– embolisation DSA

After 12 sessions of hepatic chemoembolisation, the arteriography determined high Lipiodol persistency inside the tumour and the lack of contrast–enhancing at this level (Fig. 8); CT also determined complete tumour necrosis (Fig. 8).

## Discussion

In the absence of an adequate treatment, the rate of survival in patients with hepatoma is several months (maximum 10 months). The most frequent causes of death are cachexia, gastro–intestinal bleeding, hepatic insufficiency, and in a small number of cases, spontaneous tumour rupture [6].

The most important prognostic factors are: number, size and location of hepatic tumours, portal vein thrombosis, cirrhosis and restant hepatic parenchyma dysfunction degree [7].

Hepatic transplant and hepatic resection are still the only curative methods of treatment. After tumour resection, the 5 year survival rate is 25 to 30% but in careful selected patients, it can reach 50%. [13, 15].

Chemoembolisation results are evaluated determining size, location and local tumour extends; very important is also the degree of hepatic failure (based on Child–Pugh classification). Patients from Child C group have a low survival rate because of the important hepatic dysfunction [10]. On patients who underwent surgery, the 5 year survival rate is higher than post– chemoembolisation (38% compared to 80%), especially when resected tumours were unique and smaller than 2 cm.

Referring to Child classification regarding the hepatic reserve, the chemoembolisation survival rate is higher than the one obtained after palliative, non–curative surgical intervention, on Child A patients [9, 11].

Regarding both palliative nature of interventional gesture and malignity of hepatic lesions, the overall prognosis remains reserved.

Nowadays, hepatic chemoembolisation is a modern endovascular treatment method of hepatomas developed on cirrhotic liver. For unresectable tumours with absolute contraindication for radiofrequency ablation, hepatic chemoembolisation remains the only method of treatment.
